# A Rare Blue in the Pink: Benign Blue Nevus on the Gingiva in an Eight-Year-Old Child

**DOI:** 10.7759/cureus.88264

**Published:** 2025-07-18

**Authors:** Pooja Panwar, Kalpna Chaudhry, Nitin Khanduri, Diganta Rava, Avnish Singh

**Affiliations:** 1 Pediatric and Preventive Dentistry, Seema Dental College and Hospital, Rishikesh, Rishikesh, IND; 2 Public Health Dentistry, Seema Dental College and Hospital, Rishikesh, Rishikesh, IND

**Keywords:** benign, blue nevus, melanocytes, mucosa, pediatric oral lesions

## Abstract

Oral melanocytic nevi are rare, benign tumors of unknown etiology that develop from melanocytes. The hard palate is where oral nevi most frequently occur, followed by the gingiva and buccal mucosa. Although asymptomatic, their clinical characteristics frequently necessitate a careful diagnosis of other pigmented diseases, including malignant melanoma. This report describes a rare instance of blue nevus in an eight-year-old female patient on the attached gingiva of the lower lip front region of the jaw. This case emphasizes how crucial it is to differentiate blue nevi from other melanocytic lesions using thorough clinical and histological analysis in order to guarantee an accurate diagnosis and suitable treatment, especially for children.

## Introduction

Blue nevi are clinically described as blue, gray, brown, or black solitary nodules, or histologically, they represent collections of melanocytes and melanophages in the dermis. Also termed nevus blau, it was first studied by Tieche in 1906 [1]. Nevi mostly occur on the skin and occasionally occur on mucous membranes. Oral blue nevus has a prevalence of 0.1% in the general population. There appears to be a predilection for females, with the mean age group impacted being in their third to fourth decade of life [2].

A pigmented nevus is defined clinically as an asymptomatic, well-defined, round or oval, flat or slightly elevated, and typically small lesion. The widely accepted explanation states that they originate from latent dendritic melanocytes that are locked in the dermis as a result of melanoblasts and their precursors migrating from the neural crest to the epidermis during embryologic development. They can be categorized histologically as Spitz, epithelioid, intradermal/intraneural, junctional, or compound-based [3].

The aim of this case report is to highlight the clinical features of a unique case of an oral blue nevus in an eight-year-old female pediatric patient that was found on the attached gingiva, the challenges in its diagnosis, and the vital need to distinguish it from other pigmented oral lesions to ensure a precise diagnosis and efficient treatment. Currently, there are no previously reported cases of blue nevus in the attached gingiva in the pediatric population.

## Case presentation

**Figure 1:**
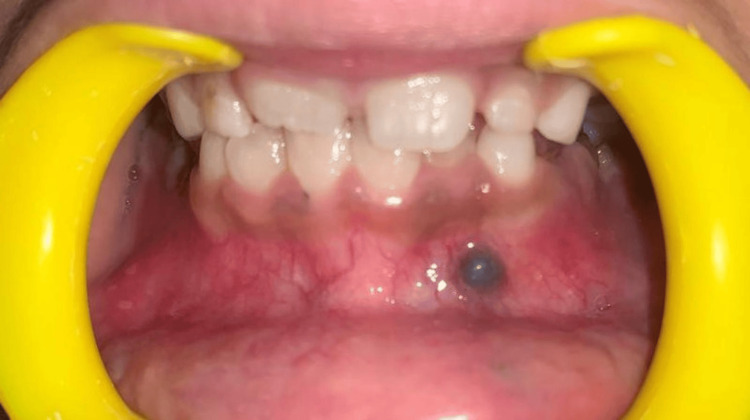
Bluish-black swelling in the left front region of the jaw

An eight-year-old female patient presented to the department of pediatric and preventive dentistry with the primary complaint of an unusual bluish-black swelling in the lower left anterior region of the mandible. The patient was apparently healthy two years prior when she noticed the bluish-black swelling in the left front region of her jaw (Figure 1). The swelling was asymptomatic, localized, elevated, and fibrous in nature. The patient reported no history of pain or fever.

**Figure 2:**
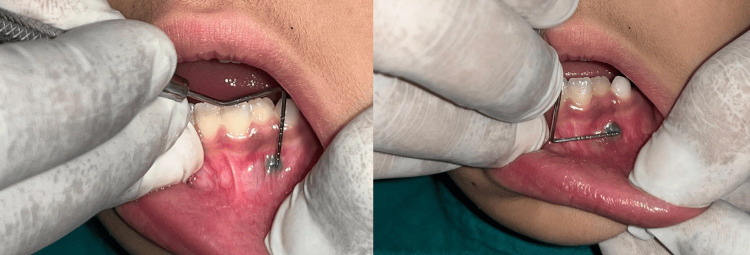
Measuring the size of the lesion using the University of Michigan "O" probe with Williams marking

On extraoral examination, facial symmetry was bilaterally maintained, no extraoral swelling was present, and regional lymph nodes were nontender and nonpalpable. The intraoral examination revealed the presence of a bluish-black nodule on the attached gingiva extending from the lower left lateral incisor (32) to the lower left canine (33) in the region, measuring 4x4 mm in size (Figure 2). On palpation, the lesion was well localized, soft to fibrous in consistency, elevated and nontender; it did not bleed on provocation, and no blanching was seen on diascopy.

The available treatment choices and the need for biopsy were explained to the patient's parent. Prior to initiating treatment, the patient's parents were thoroughly briefed on the entire process, and informed consent was obtained. The parents' consent was obtained for the publication of the case report.

A provisional diagnosis of hemangioma was made. The differential diagnosis included melanocytic macule, fibroma, and malignant melanoma.

Case management

**Figure 3:**
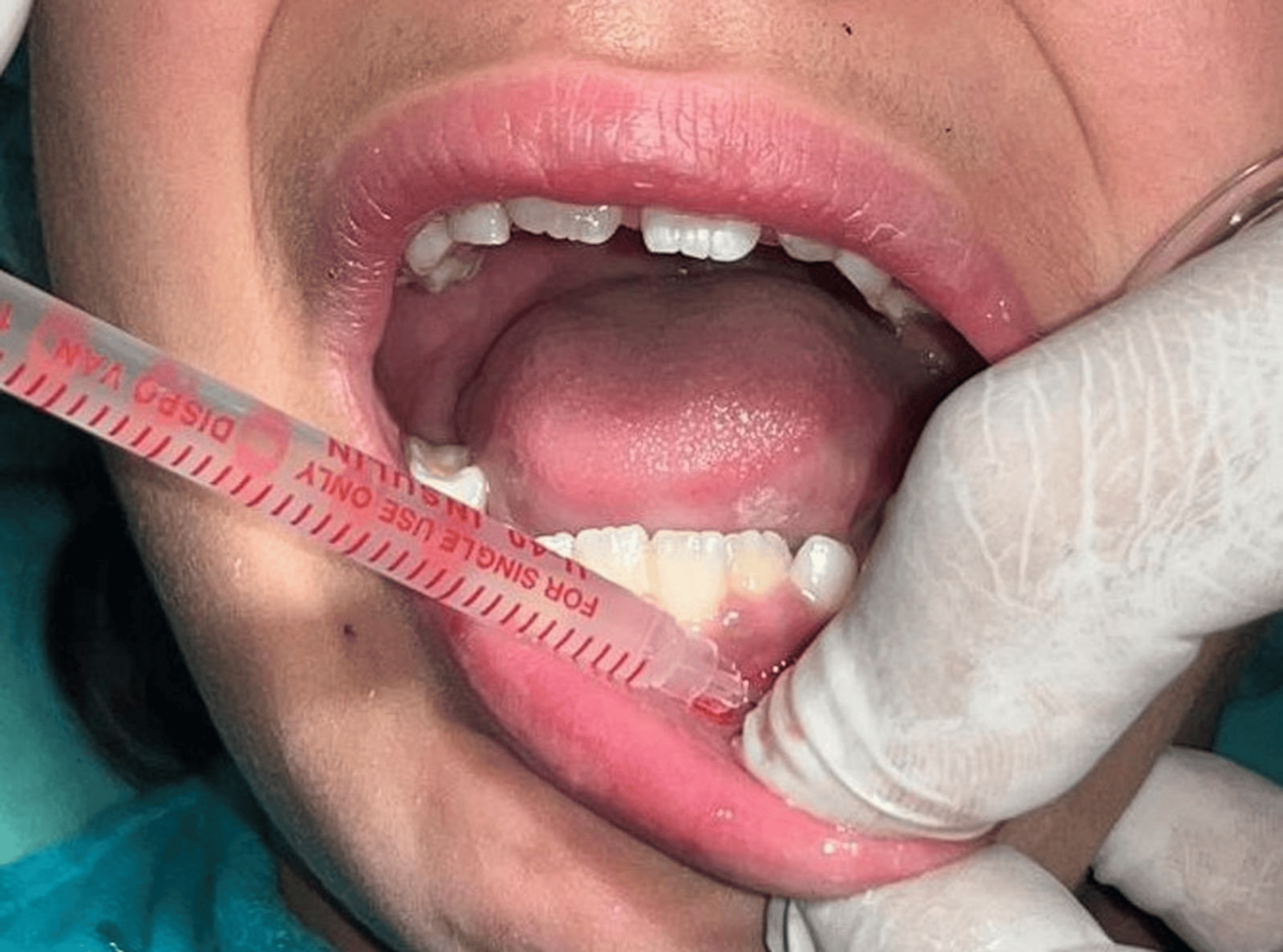
Administration of local anesthesia

**Figure 4:**
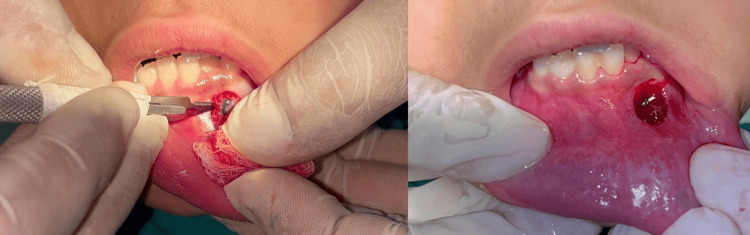
Surgical excision of the lesion using a scalpel

**Figure 5:**
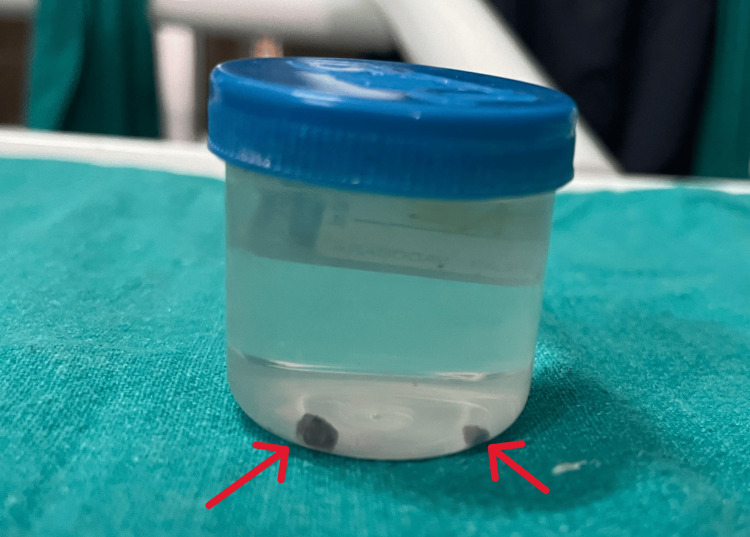
Biopsy sample collected in a sterile container

**Figure 6:**
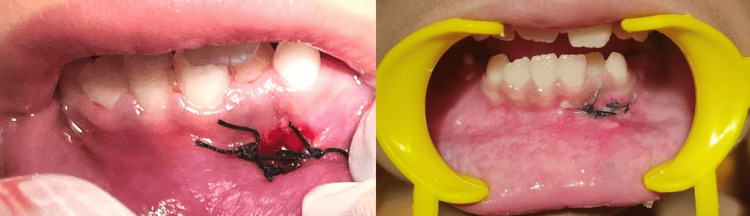
Simple interrupted sutures placed (3-0 silk braided)

**Figure 7:**
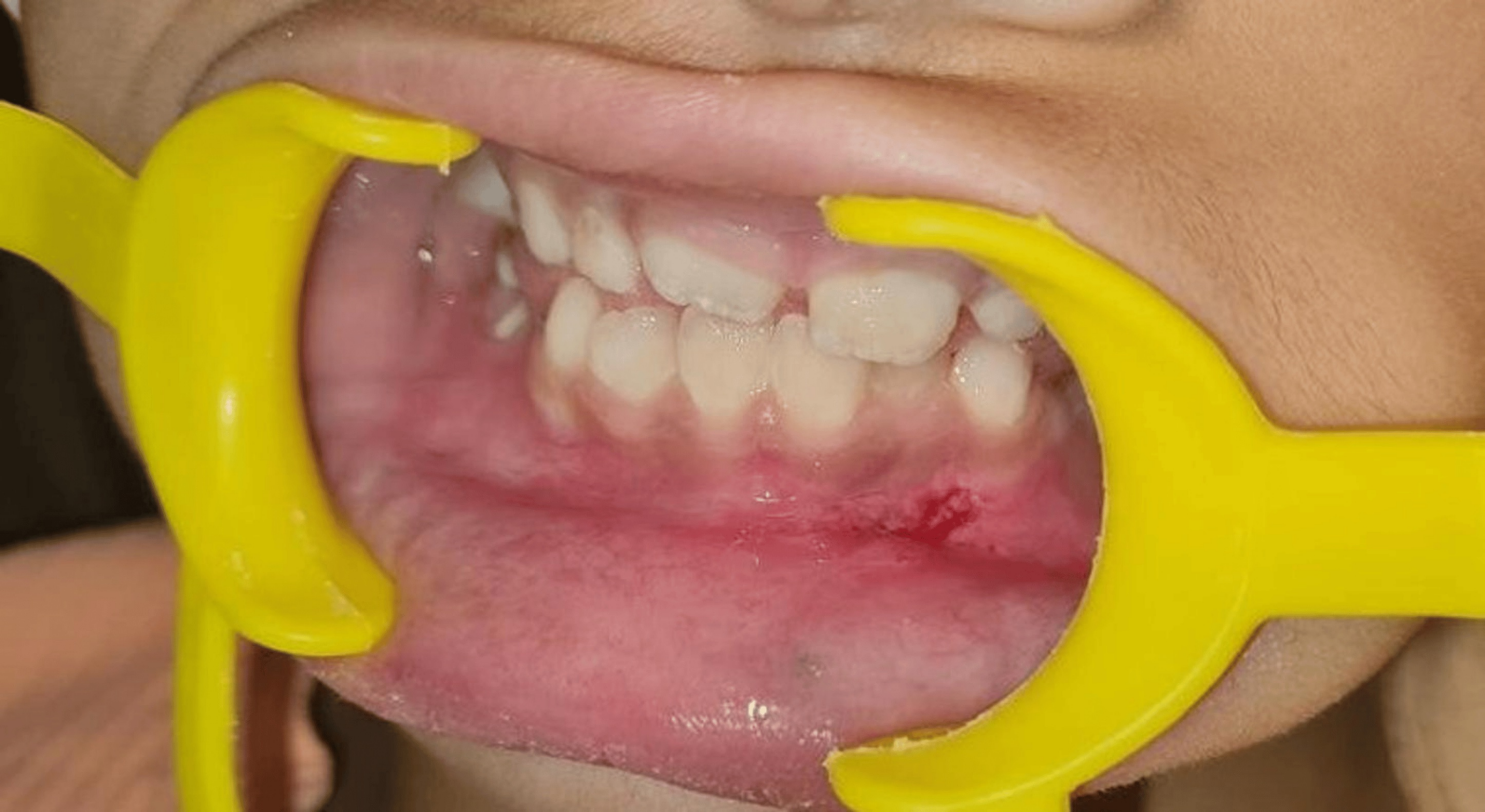
Postoperative healing after one week

After administration of local anesthesia (lidocaine 2% with epinephrine 1:80000) (Figure 3), surgical removal of the lesion was done using a scalpel (Figure 4). It was stored in a sterile container for biopsy purposes (Figure 5). Immediate postoperative care was provided, and simple interrupted sutures were placed (3-0 silk braided) (Figure 6). The biopsy sample was stored in a sterile container using a fixative-to-tissue volume ratio of at least 10:1. The excised tissue was immediately immersed in 10% neutral buffered formalin (4% formaldehyde) and was then sent for histopathological examination. One week postoperatively, the healing was satisfactory (Figure 7).

Histopathological findings

**Figure 8:**
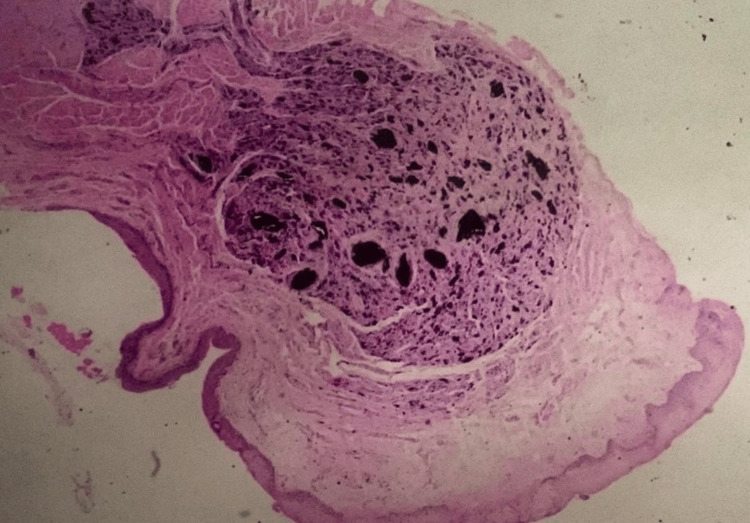
Histopathological image stained with hematoxylin and eosin, showing nests of nevus cells within the connective tissue beneath the oral epithelium

The histologic section demonstrated a wedge-shaped proliferation of bland fusiform and dendritic melanocytes with abundant pigment and melanophages dissecting dermal collagen. The histopathological diagnosis indicated a benign blue nevus (Figure 8).

**Figure 9:**
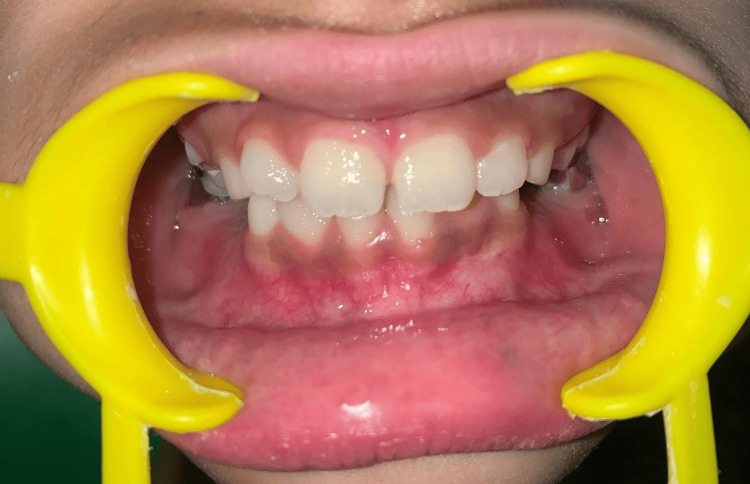
Follow-up after one year

A one-year follow-up revealed no signs of recurrence, demonstrating a favorable clinical outcome (Figure 9).

## Discussion

Pigmented lesions in the oral cavity are frequently asymptomatic and are often discovered incidentally during routine examinations; thus, diagnosing them can be challenging. The majority of pigmented lesions require surgical excision for further histopathological analysis, even though the ABCDE (Asymmetry, Border, Color, Diameter, Evolution) system can be used as a preliminary tool for assessing the malignant potential of oral lesions; clinical evaluation alone is insufficient for a conclusive diagnosis. Consequently, the majority of pigmented lesions necessitate surgical excision for subsequent histopathological examination [1,4].

The probability of clinical variance occurrence is 55% in intramucosal type, followed by common blue nevus at 32%, compound nevi at 6%, junctional nevi at 5%, and combination nevi at 2%, according to a review of several cases conducted by Buchner and Hansen [5]. According to published research, the hard palate accounts for 42% of oral nevi, followed by the buccal mucosa at 17% and the gingiva at 8% [6]. Given that malignant melanoma, melanocytic macule, hemangioma, and fibroma are the differential diagnoses for focally pigmented lesions, we have included their distinguishing clinical and histopathological features to strengthen the diagnostic rationale of this case report and better support our final diagnosis of intraoral benign blue nevus. (1) A malignant melanoma was ruled out due to the absence of cytologic atypia and an infiltrative growth pattern. The nevus cells were well circumscribed, uniform, and lacked malignant features. (2) Melanocytic macules typically present clinically as flat, uniformly pigmented lesions. Histologically, they show increased melanin in the basal cell layer without nevus cell proliferation in the connective tissue; hence, this diagnosis was excluded. (3) Hemangioma was considered due to the bluish clinical appearance. However, it was excluded based on the lesion's firm consistency, as hemangiomas are typically soft and compressible, and the absence of blanching on diascopy. (4) Fibroma was also considered due to the lesion's firm nature. However, fibromas are typically dome-shaped, pale, or mucosa-colored and arise from chronic irritation or trauma. Histologically, they consist of dense collagenized connective tissue without nevus cells or pigmentation. In contrast, our lesion showed pigmented nevus cells in nests in the connective tissue stroma, confirming a melanocytic origin.

Therefore, biopsy becomes essential for correctly diagnosing oral melanocytic nevus. For these oral lesions, conservative surgical excision is still the recommended course of treatment [7].

In the present case, we continued to follow up with the patient every three months for a whole year. In contrast to a case report by Ramesh et al., where the patient at one-year follow-up revealed a tiny area of focal hyperpigmentation at the location of the prior lesion [8], no recurrence was observed after one year of follow-up in our patient (Figure 9), and full healing is acknowledged.

Although some studies contradict this assertion, nevi of the mucous membrane have been found to have the most potential for malignant transformation into melanoma [9]. Therefore, it is advisable to identify any pigmented lesions as soon as possible due to the malignant potential of blue nevi and the occurrence of pigmented macules in roughly one-third of patients with preliminary oral melanoma [10].

## Conclusions

Despite being frequently benign, oral pigmented lesions require careful examination due to their potential for malignancy. This rare instance of a benign blue nevus on the pediatric patient's attached gingiva emphasizes the value of early biopsy, histological confirmation, and clinical vigilance. Prompt diagnosis and surgical excision are essential for effective management and to rule out malignant conditions like melanoma.
